# Delayed‐onset heparin‐induced thrombocytopenia complicated with saddle embolus

**DOI:** 10.1002/ccr3.6085

**Published:** 2022-07-19

**Authors:** Faezeh Sadat Naji, Mahan Shafie, Mahbod Issaiy, Narjes Zarei Jalalabadi, Samaneh Parsa

**Affiliations:** ^1^ Department of Internal Medicine, Imam Khomeini Hospital Complex Tehran University of Medical Sciences Tehran Iran; ^2^ School of Medicine Tehran University of Medical Sciences Tehran Iran

**Keywords:** heparin‐induced thrombocytopenia, platelet factor 4, saddle embolus, thrombosis

## Abstract

Delayed‐onset heparin‐induced thrombocytopenia is a rare complication of heparin in which thrombocytopenia and thrombosis occur several days after heparin cessation. We describe a 47‐year‐old female patient with a history of melanoma and multiple surgeries presented to the emergency department with acute dyspnea and chest pain on the eighth day after discharge.

## Abstract 2

Delayed‐onset heparin‐induced thrombocytopenia should be considered whenever a patient presents with thrombosis with a history of hospitalization even after several days. A history of probable heparin exposure should be searched more precisely.

## 1 INTRODUCTION

Heparin‐induced thrombocytopenia (HIT) is a rare condition leading to an increased risk of thrombosis despite thrombocytopenia. It is estimated to have a 1–5% incidence among heparin‐treated patients.^1^ HIT can occur with both unfractionated (UFH) and low molecular weight heparin (LMWH); however, the former is 2–5 times more frequent than the latter.^2^ It is known as an immune‐mediated disease in which antibodies against the complexes of platelet factor 4 (PF4) and heparin are formed, leading to platelet aggregation.^2^, ^3^ Treatment of the patients with HIT is of utmost importance, owing to the high mortality rate among untreated patients (up to 20%) which can be significantly narrowed by early treatment (<2%).^4^ Delayed‐onset HIT is a subtype of HIT in which thrombocytopenia and thrombosis occur several days after discontinuation of heparin, and it is believed to be caused by circulating immunoglobulin G antibodies.^5^ In the following case report, we present a lady who developed thrombocytopenia and saddle embolus in the main pulmonary artery several days after surgery and received prophylactic heparin.

## 2 CASE REPORT

A 47‐year‐old lady, a known case of malignant melanoma, who had undergone several surgeries and was recently discharged following selective lymph node dissection and left jugular vein ligation, presented complaining of dyspnea and pleuritic chest pain on the eighth day after discharge. Physical examination showed that she was afebrile, and her blood pressure was 105/60 mmHg with a pulse rate of 114 beats per minute. Her respiratory rate was 27 respirations per minute with a partial oxygen saturation of 94% using a finger pulse oximeter. Lung auscultation was unremarkable. A neck examination revealed an area suggestive of hematoma next to the site of her recent surgery. On primary evaluation, we noticed severe thrombocytopenia with a platelet count of 12 × 10^9^/L. More laboratory findings are presented in Table 1.

**TABLE 1:** Laboratory values at the day of admission

Test	Value	Unit	Normal range
WBC	11.1	×1000/mm^3^	4.1–10.1
Hb	10.2	g/dl	12–16
Plt	12,000	×1000/mm^3^	150–400
ESR	33	mm/h	<20
CRP	58	mg/L	<5
Troponin I	<0.2	ng/ml	<0.29
NT‐PRO‐BNP	81.2	pg/ml	<125
PT	14.3	Sec	11–15
PTT	26	Sec	25–40
INR	1.17	–	1–1.4
D‐dimer	>10	mg/L	<0.55
Fibrinogen	381	mg/dl	200–400
Urea	32	mg/dl	15–50
Creatinine	0.8	mg/dl	0.7–1.4
LDH	773	U/L	140–280

Abbreviations: CRP, c‐reactive protein; ESR, erythrocyte sedimentation rate; Hb, hemoglobin; INR, international normalized ratio; LDH, lactate dehydrogenase.; Plt, platelets; PT, prothrombin time; PTT, partial thromboplastin time; WBC, white blood cell.

Reviewing her recent medical records showed that the patient's platelet count was 186 × 10^9^/L preoperatively without any signs of blood loss. Her discharge had been planned by the surgery service and 7 days after discharge, she experienced acute‐onset dyspnea and pleuritic chest pain leading to her readmission. Pulmonary thromboembolism was strongly suggested according to Wells criteria and was confirmed by pulmonary CT angiography revealing a saddle embolus visualized as a filling defect at the site of pulmonary artery bifurcation. The embolus extended to the superior and inferior lobar branches and their segmental arteries on the right side and some of the segmental branches of superior and inferior lobes on the left side. Superficial neck ultrasonography displayed bilateral subacute to acute thrombosis of jugular veins and heterogeneous hyperechoic lesion with the size 110 × 34 × 27 mm suggestive of hematoma. Furthermore, lower extremities Doppler ultrasonography revealed acute deep vein thrombosis (DVT) in the left popliteal vein.

After excluding thrombotic thrombocytopenic purpura (TTP), disseminated intravascular coagulopathy (DIC), drug‐induced thrombocytopenia, thrombocytopenia associated with severe sepsis and transient mild postoperative thrombocytopenia, simultaneous platelet transfusion, and heparin infusion at a reduced rate was considered. There was no evidence of schistocytes in peripheral blood smears, which makes TTP and DIC unlikely, nor did it meet other laboratory and clinical criteria for TTP and DIC. There was no other drug history leading to thrombocytopenia and also sepsis was not considered for the patient due to negative SIRS, negative cultures, and negative clinical findings. Postoperative thrombocytopenia is usually associated with a mild and transient decrease in platelets, which was not relatable to our case. Eventually, there was no history of blood or platelet transfusion. A consultation with an interventional radiology service was done regarding surgical pulmonary embolectomy or catheter‐directed thrombolysis; however, the patient was not eligible for either of them.

Two days later, despite platelet transfusion, the patient's platelet count dropped even further, which encouraged the suspicion of heparin‐induced thrombocytopenia (HIT). A retrograde review of the patient's medical records revealed that she had received unfractionated heparin (UFH) as thromboprophylaxis on her previous admission for neck surgery. Having calculated more than 6 points in 4T‐score made the diagnosis of HIT highly probable. An ELISA test for PF4‐heparin antibodies was performed which resulted significantly positive. Heparin administration was immediately halted, and rivaroxaban was started at the dose of 15 mg twice daily as the alternative treatment. On the second day of rivaroxaban administration, her platelet count increased to 87 × 10^9^/L and within 5 days, the platelet count was normalized. Besides, resolution of the neck hematoma was documented by serial ultrasound studies.

## 3 DISCUSSION

In this report, we presented a lady who underwent neck surgery and despite having received prophylactic unfractionated heparin, developed massive emboli in three different vascular systems: pulmonary artery, jugular veins, and popliteal vein. These embolic events coinciding with severe thrombocytopenia led us to the diagnosis of HIT. There were several challenges regarding the patient's diagnosis and treatment, including the severe and extensive thromboembolic events, which are not typical of HIT. At presentation, pulmonary thromboembolism was thought to be caused by the patient's underlying malignancy and her recent surgery. The thrombosis of jugular veins was attributed to her recent neck operation which involved manipulation of the local veins. Furthermore, due to the incompleteness of electronic medical records, at first, the treatment team was not aware of the patient's baseline platelet count. After excluding TTP and DIC, thrombocytopenia was thought to be caused by bone marrow metastasis or myelodysplastic syndrome (MDS).

Entering the bloodstream, heparin will be immediately bound to platelet factor 4 (PF4). This complex triggers the production of some antibodies, IgG and IgM, which attach to the heparin‐PF4 complex. This attachment will activate the platelets to do three main actions; producing more PF4, aggregation, and premature elimination. The first action accelerates this cycle, the second causes susceptibility to thrombosis, and the last one is the reason for thrombocytopenia.^2^, ^3^, ^6^ Most commonly, clinical manifestations of HIT start with thrombocytopenia and later are complemented with thrombotic events (mostly deep vein thrombosis and pulmonary embolism). Usually, a decrease of over 50% in the platelet count is apparent within 4–10 days after the first exposure to heparin.^6^ Using unfractionated heparin (rather than LMWH), undergoing a surgical procedure (rather than medical and obstetric patients), being over 40 years old, and female gender are the most known risk factors of HIT.^7^ Thromboembolic complications can involve veins, arteries, or both and include deep venous thrombosis, pulmonary embolism, myocardial infarction, thrombotic stroke, and occlusion of limb arteries.^8^

There are two types of HIT syndromes. HIT type I is characterized by a clinically non‐significant, benign, and transient drop in platelet count due to a non‐immune platelet aggregation mechanism and is not associated with an increased risk of thrombosis. This form of HIT occurs within the first 2 days of heparin administration and a normal platelet count is expected without the need to discontinue heparin. The second form of HIT, HIT type II, is a clinically significant syndrome with immune‐mediated pathogenesis caused by antibodies directed against complexes containing heparin and platelet factor 4 (PF4).^9^ Thrombosis is an important manifestation of this type of HIT which occurs in up to 25 percent of patients with thrombocytopenia and along with thrombosis.^10^ Delayed‐onset heparin‐induced thrombocytopenia is a variant of HIT in which thrombocytopenia and/or thrombosis occur at least 5 days after heparin withdrawal.^11^ However, there exists another definition for delayed‐onset HIT proposed by Rice et al.^5^ They suggested that delayed‐onset HIT has to be considered whenever a patient with prior exposure to heparin and its derivatives presents to the hospital with HIT‐specific complications such as thromboembolism. The latter definition encompasses a wider range of patients as it does not constrain it to a time limit. In two previous reports, this phenomenon was observed as two patients presented with massive bilateral pulmonary emboli; one of them well after their heart surgery.^12^, ^13^

The diagnostic criteria include both clinical and biochemical aspects.^14^ One of the most popular clinical scoring systems for HIT diagnosis is the 4T score, which scores a multitude of important clinical factors including thrombocytopenia, the timing of the platelet count drop, clinical sequelae like thrombosis, and the likelihood of other causes for thrombocytopenia. The 4T score is a reliable system to rule out HIT due to its high negative predictive value; However, its positive predictive value is not high enough to confirm the diagnosis.^4^ Clinical features alone are not sufficient to diagnose HIT with certainty. The gold standard laboratory tests for confirming diagnosis are two platelet‐activation assays: heparin‐induced platelet aggregation test (HIPA) and serotonin‐release assay (SRA).^15^ After the confirmation of HIT diagnosis, heparin administration must be immediately discontinued. Heparin and its derivatives could increase the risk of thrombosis, therefore, non‐heparin anticoagulant agents, such as direct thrombin inhibitors, should be initiated for the patient.^6^

In conclusion, we suggest that HIT, especially delayed‐onset HIT, should be considered whenever a patient presents with thrombosis and concurrent thrombocytopenia even with a prior suspicious history of heparin exposure. Particularly, due to the fact that unlike treatment options for other thromboembolic conditions, HIT complications necessitate immediate discontinuation of heparin and its derivatives.

## AUTHOR CONTRIBUTIONS

FN contributed in developing the research idea, composing, and revising the manuscript. MS contributed in developing the research idea, composing, and revising the manuscript. MI contributed in composing and revising the manuscript. NZ contributed in composing and revising the manuscript. SP contributed in developing the research idea and revising the manuscript.

## CONFLICT OF INTEREST

The authors have no conflict of interest to declare.

## ETHICAL APPROVAL

This study was approved by the research and ethics committee of Tehran University of Medical Sciences. The patient has given her informed consent to publish this case.

## CONSENT

Written informed consent was obtained from the patient for publication of this case report and any accompanying images. A copy of the written consent is available for review by the Editor‐ in‐Chief of this journal.

## Data Availability

Data sharing is not applicable to this article as no datasets were generated or analyzed during the current study.
